# A Case of Placental Presentation

**Published:** 1887-01

**Authors:** S. L. Lowry

**Affiliations:** San Antonio, Texas


### A CASE OF PLACENTAL PRESENTATION.

*By S. L. Lowry A. M., M. D., Sau Antonio, Texas.*

For Daniels Texas Medical Journal.

I WAS hurriedly called on the morning of 28th inst. to see Mrs. C. who, I was informed, was threatened with miscarriage, and was suffering very great pain. On reaching the house, I found a woman, seven months pregnant, of rather small size and frail appearance, lying in bed, and complaining of a very intense, constant, heavy, aching pain in the right lumbar region. On inquiry I learned that she had been in good health until the beginning of the present attack, which came on at 1 o'clock of the night just past, and had persisted up to the present time. On examination, the os uteri was found in a nomal condition, and there were no indications that uterine contractions had begun. The thermometer registered 102° in the axilla, the bowels had been moved during the night, but the urine had not been passed for twelve hours. I was exceedingly puzzled to know the cause of the pain. After removing the urine with a catheter and preserving it for further examination, I administered a hypodermic of morphine, and ordered a bromide and chloral mixture to be taken until I should return in the afternoon. About 5 o'clock I was hastily summonsed again, and was told by the messenger that instead of being relieved, she had steadily grown worse since my last visit. I hastily repaired again to the house, and found my patient seemingly suffering the most excruciating pain. Its character and location were the same as had been described in the morning, but the intensity seemed greatly increased. Her temperature had arisen to 105° and her small thready pulse, blanched appearance, and depressed condition seemed to portend immediate dissolution. I administered another hypodermic of morphine, and after waiting a few minutes for its quieting effect, I proceeded to make a second vaginal examination. I found the os soft and dilatable but not open, and on gently insinuating my finger through the internal os, I was startled by a sudden rush of blood, which flowed freely from the uterus until fully a pint had been lost. In a minute the hemorrhage had partially ceased, and on further examination I now for the first time discovered that I had a case of *Placenta Proevia* to deal with. I immediately informed the husband of the gravity of the situation, and summoned medical aid in the person of Dr. H. D. Barnitz, who made an examination, and confirmed my diagnosis. After the administration of chloroform by Dr. B., I inserted my right hand, well oiled, into the vagina, and after gradually dilating the os uteri with my fingers, and detaching the placental attachments on one side, I succeeded in introducing my hand into the uterus, and grasping both feet of the child between my fingers, I soon brought down its body, and delivered the woman in a few minutes and with very little loss of blood. Reintroducing my hand, I loosened the remaining placental attachment, and brought away the after-birth complete. On recovering from the chloroform, she seemed in comparative comfort, and I left her, after ordering five grains of quinine every four hours, and administering half drachm of Fluid Extract of Ergot hypodermically. At my next visit her temperature was normal, and I now consider her out of danger, and in a fair way to a speedy recovery.

I report the case not only on account of the comparative rarity of such cases, and the danger attending them; but more particuarly on account of the very severe pain, and high fever, which attended it, thus presenting two dangerous, and unlooked-for complications.

The extreme pain and great prostration were probably due to the loss of blood, and the pressure caused by its retention in an over-distended uterus; but why the pain should be referred to the right lumbar region I am unable to determine. Whether the rise of fever was due to the same cause, or some other complication I leave to the better judgment of those who have had more experience in such cases than myself.

